# The functional loss of the retinoblastoma tumour suppressor is a common event in basal-like and luminal B breast carcinomas

**DOI:** 10.1186/bcr2142

**Published:** 2008-09-09

**Authors:** Jason I Herschkowitz, Xiaping He, Cheng Fan, Charles M Perou

**Affiliations:** 1 Lineberger Comprehensive Cancer Center, University of North Carolina, Chapel Hill, NC 27599, USA; 2 Department of Genetics, University of North Carolina, Chapel Hill, NC 27599, USA; 3 Department of Pathology & Laboratory Medicine, University of North Carolina, Chapel Hill, NC 27599, USA; 4 Department of Molecular and Cellular Biology, Baylor College of Medicine, One Baylor Plaza, DeBakey M635, Houston, TX 77030, USA

## Abstract

**Introduction:**

Breast cancers can be classified using whole genome expression into distinct subtypes that show differences in prognosis. One of these groups, the basal-like subtype, is poorly differentiated, highly metastatic, genomically unstable, and contains specific genetic alterations such as the loss of tumour protein 53 (TP53). The loss of the retinoblastoma tumour suppressor encoded by the RB1 locus is a well-characterised occurrence in many tumour types; however, its role in breast cancer is less clear with many reports demonstrating a loss of heterozygosity that does not correlate with a loss of RB1 protein expression.

**Methods:**

We used gene expression analysis for tumour subtyping and polymorphic markers located at the RB1 locus to assess the frequency of loss of heterozygosity in 88 primary human breast carcinomas and their normal tissue genomic DNA samples.

**Results:**

RB1 loss of heterozygosity was observed at an overall frequency of 39%, with a high frequency in basal-like (72%) and luminal B (62%) tumours. These tumours also concurrently showed low expression of RB1 mRNA. p16^INK4a ^was highly expressed in basal-like tumours, presumably due to a previously reported feedback loop caused by RB1 loss. An RB1 loss of heterozygosity signature was developed and shown to be highly prognostic, and was potentially a predictive marker of response to neoadjuvant chemotherapy.

**Conclusions:**

These results suggest that the functional loss of RB1 is common in basal-like tumours, which may play a key role in dictating their aggressive biology and unique therapeutic responses.

## Introduction

The retinoblastoma tumour suppressor gene (RB1) encodes a nuclear phosphoprotein that plays a central role in regulating the cell cycle [1]. Inactivation of both alleles of this gene is involved in the development of retinoblastoma, which is a rare childhood malignancy. The loss of RB1 is also a well-characterised occurrence in many other human tumour types and it is probable that the p16^INK4a^-CDK4/6-RB pathway is disrupted in most human tumours [2]. RB1 regulates progression through the G1 to S-phase transition of the cell cycle. In cells entering the cell cycle, extracellular signals induce the expression of D-type cyclins, which bind to and activate cyclin-dependent kinases (CDK4 and CDK6); these complexes in turn lead to the phosphorylation of RB and its dissociation from E2F family members that then transcriptionally activate many genes required for the S phase [1]. The INK4 family of CDK inhibitors (p16^INK4a^, p15^INK4b^, p18^INK4c ^and p19^INK4d^) inhibits CDK4 and CDK6, retaining RB in its hypo-phosphorylated E2F-associated state, thereby preventing G1 to S-phase progression. It has recently been shown that CDK4 and CDK6 (and CDK2) are dispensable for driving the essential cell cycle; however, they are required in specialised tissues and possibly to achieve higher levels of proliferation [3].

Inactivation of the RB1 gene in breast cancer was originally shown using a series of cell lines [4]. Subsequently, loss of heterozygosity (LOH) has been observed in primary tumours, but does not necessarily correlate with low RB1 protein expression as assessed by immunohistochemistry [5,6]. LOH has, however, been shown to correlate with low RB1 mRNA expression [5]. There are also genetic events upstream of RB1 that may be present in breast tumours, which can negatively impact RB1 function by promoting its phosphorylation, that include p16^INK4a ^loss [7] and cyclin D1 amplification/overexpression [8].

Breast cancer is a heterogeneous disease, which can be separated into clinically significant subtypes as defined by molecular profiling [9,10]. In addition to reproducible gene expression differences between these subtypes, specific molecular alterations continue to be identified that correlate with each subtype. Tumours of the basal-like subtype generally have a high mitotic index, tend to be p53 mutated [11] and highly express the proliferation signature, which is a gene cluster shown to contain many E2F target genes [12,13]. Here we report that LOH at the RB1 locus occurs at a high frequency in human basal-like and luminal B tumours, while occurring infrequently in luminal A and human epidermal growth factor receptor 2 (HER2)-enriched tumours. p16^INK4a ^is also highly expressed both by microarray and by immunohistochemistry in most of the RB1 LOH basal-like tumours, presumably due to a feedback caused by RB1 loss. These results further illustrate the unique biology of each breast cancer subtype.

## Materials and methods

### Patient samples and breast cancer microarray data sets

All human tumour samples were collected from fresh frozen primary breast tumours using protocols approved by the Institutional Review Board and were profiled as described previously using Agilent (Agilent Technologies, United States) oligo microarrays [9,14-16]. The primary microarray data for the 232 sample data set is available in the Gene Expression Omnibus (GEO) [GEO:GSE3165]. The data set containing only tumours with informative LOH status can be found in GEO [GEO:GSE10884], with 13 new samples in this study.

### DNA isolation and detection of RB1 loss of heterozygosity

Patient DNA from lymphocytes, normal breast tissues and breast tumours was isolated using the DNeasy kit (Qiagen, Germany). We used two polymorphic markers; a variable number tandem repeat (VNTR) in intron 20 and D13S153; a microsatellite marker for RB1 LOH analyses. The primers were previously published for intron 20 [17]. The primers for D13S153 [AFM058xd6a, AFM058xd6m] were obtained from the Genome Data Bank (Johns Hopkins University, Baltimore, MD) [18]. The PCR products were run on the Agilent Bioanalyzer using DNA 1000 kit (Agilent, United States). The patient was called informative when there were two alleles present in their normal DNA. LOH was called when there was at least a 50% loss of an allele/band in the tumour for at least one of the two polymorphic markers.

### Statistical analysis

The chi-square test and Fisher-Freeman-Halton exact test were used to examine correlations between RB1 LOH status, immunostaining and tumour subtype using SAS 9.1 (Cary, NC). An analysis of variance (ANOVA) and unpaired Student's t-test were performed and a box plot graph plotted to compare RB1 LOH status or immunostaining with gene expression using web based 'Statistics to Use' [19]. A Significance Analysis of Microarrays (SAM) was performed to identify genes that were significantly differentially expressed between tumours with RB1 LOH-positive tumours compared with LOH-negative tumours [20]. Expression analysis systemic explorer was used to identify gene ontology categories overrepresented in the RB LOH gene list compared with the genes present on the array.

Whole genome RVista was used to determine the known transcription factor binding sites overrepresented in the 1 kb upstream region in the lists of genes examined compared with the rest of the RefSeq genes in the whole human genome [21]. Hypergeometric mean analysis was performed as described by Chung and colleagues [22]; this comparison gives the likelihood of finding co-occurrences between these gene sets by chance. The simulation was performed independently for each pair of gene sets analysed.

### Immunohistochemistry

Formalin-fixed, paraffin-embedded tissue sections (approximately 5 μm) were processed using standard immunostaining methods. Following deparaffinisation in xylenes, slides were rehydrated through a graded series of alcohol and rinsed in PBS. Endogenous peroxidase activity was blocked with 3% hydrogen peroxidase. Samples were steamed for antigen retrieval with 10 mM citrate buffer (pH 6.0) for 30 minutes. Slides were then incubated for 20 minutes with diluted normal blocking serum. The sections were incubated for 60 minutes at room temperature with primary antibody pRb (Visionbiosystems Novocastra, NCL-L-RB-358 clone 13A10, 1:50 dilution) or p16 (Santa Cruz, H-156, 1:50 dilution). The slides were incubated for 45 minutes with diluted biotinylated secondary antibody (1:250 dilution) and 30 minutes with Vectastain Elite ABC reagent (Vector Laboratories, United States). Sections were incubated in peroxidase substrate solution for visualisation. Slides were counterstained with haematoxylin and examined by light microscopy. Tumour immunoreactivity was scored as: 0 = negative, 1 = weak positive, 2 = moderate positive and 3 = strong positive. The evaluation of p16^INK4a ^and RB1 protein staining was performed by two blinded independent researchers.

## Results

### Basal-like tumours show low expression of the RB1 transcript

**Figure 1:**
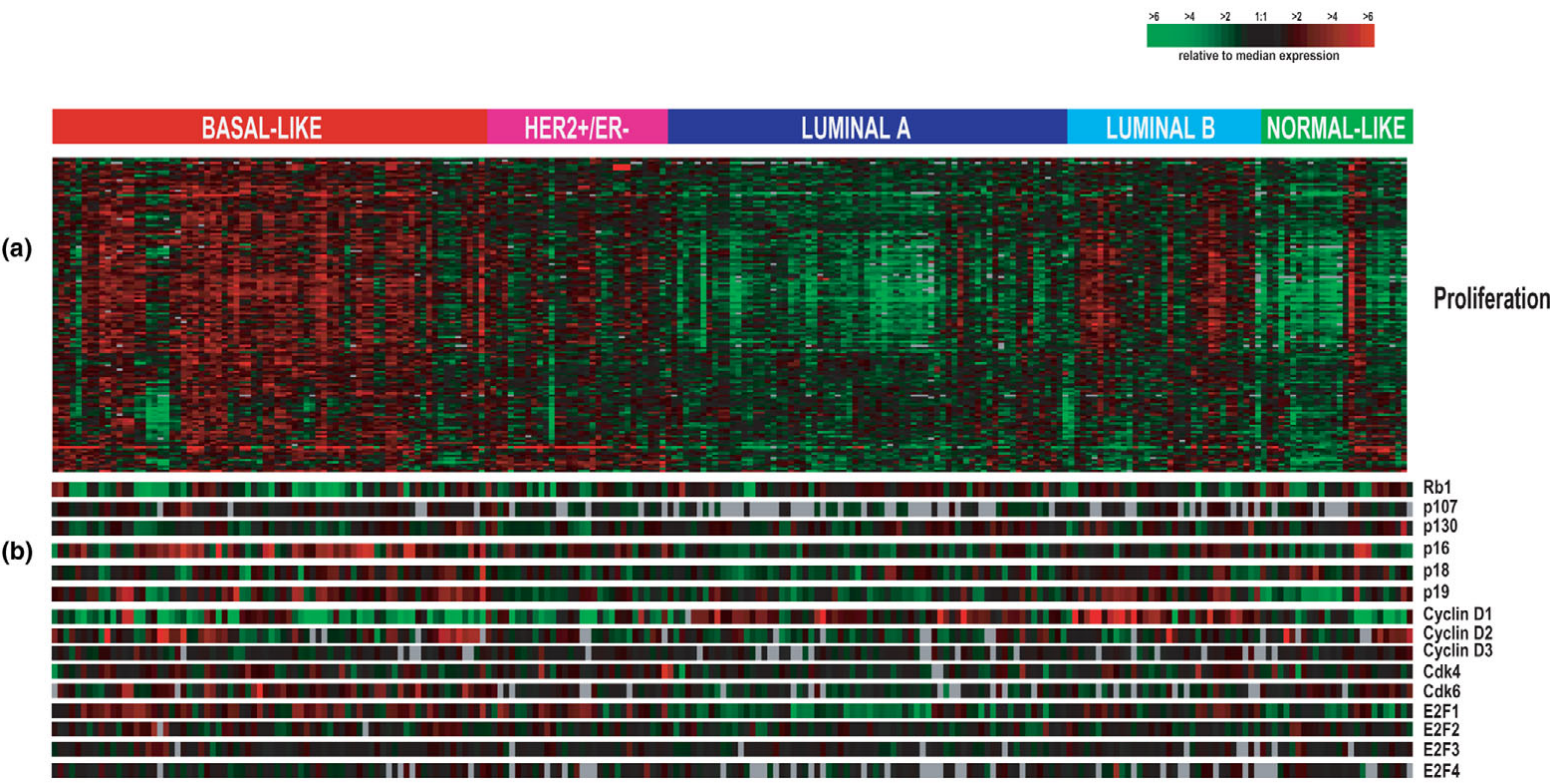
**The expression of retinoblastoma pathway members varies across breast cancer intrinsic subtypes**. Two-hundred and thirty-two human samples are ordered by subtype according to the five-class single sample predictor from Hu and colleagues [9]. Samples are coloured according to their subtype: red = basal-like, dark blue = luminal A, light blue = luminal B, pink = human epidermal growth factor receptor 2 (HER2)-enriched and green = normal breast-like. (a) proliferation gene cluster. (b) Retinoblastoma (RB)-pathway genes which are present on the array and passed data quality filtering criteria of showing a signal intensity of more than 30 units in both channels.

**Figure 2:**
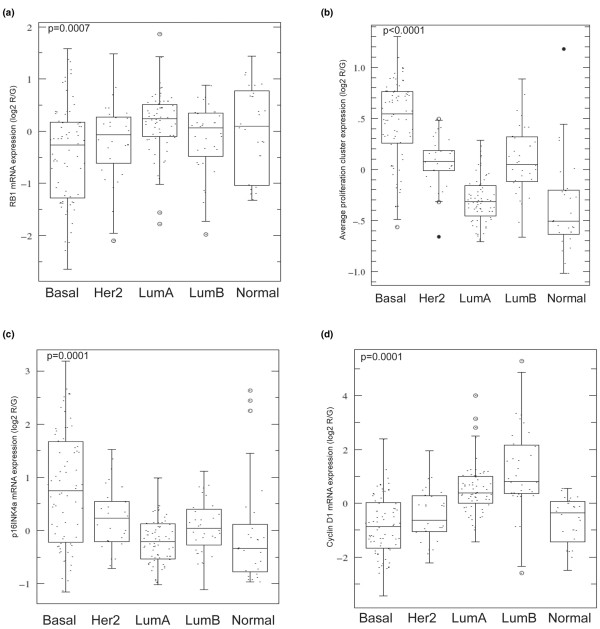
**The expression of RB1, p16^INK4a ^and cyclin D1 varies across the breast cancer intrinsic subtypes**. Box plot comparisons of (a) Retinoblastoma (RB) 1, (b) proliferation signature, (c) p16^INK4a ^and (d) cyclin D1 mRNA expression relative to the five intrinsic subtypes as defined by the five-class centroid predictor.

Tumours of the basal-like subtype have been shown in several studies to have a high mitotic rate and to highly express a proliferation gene signature [11,12]. For this reason, we postulated that there might be a defect in the RB pathway in these tumours. We first examined the expression levels of the core components and regulators of the RB pathway in a previously published microarray data set [9,14] that contained 232 microarrays consisting of 184 primary breast tumour samples and nine normal breast samples (Figure 1). On average, RB1 was expressed at the lowest levels in basal-like tumours and at the highest levels in luminal A tumours (Figures 1 and 2a), while the converse was observed for the average expression of a previously defined proliferation gene signature [9] (Figure 2b). Tumours of the basal-like subtype also frequently showed high levels of p16^INK4a ^(Figures 1 and 2c) and E2F1 (Figure 1 and data not shown). Cyclin D1 levels, on the other hand, were elevated mainly in luminal tumours including noticeably higher expression in many luminal B tumours (Figures 1 and 2d); in addition, it was recently reported that there are often high level gains of the 11q13 locus that includes cyclin D1 in luminal tumours [23], therefore suggesting that cyclin D1 high expression can be considered a 'luminal event'. Similar findings concerning RB1 mRNA expression and the basal-like subtype have also recently been reported suggesting that this is a reproducible expression feature of basal-like tumours [24].

### LOH at the RB1 locus is associated with high proliferation rates and tumour subtype

**Figure 3:**
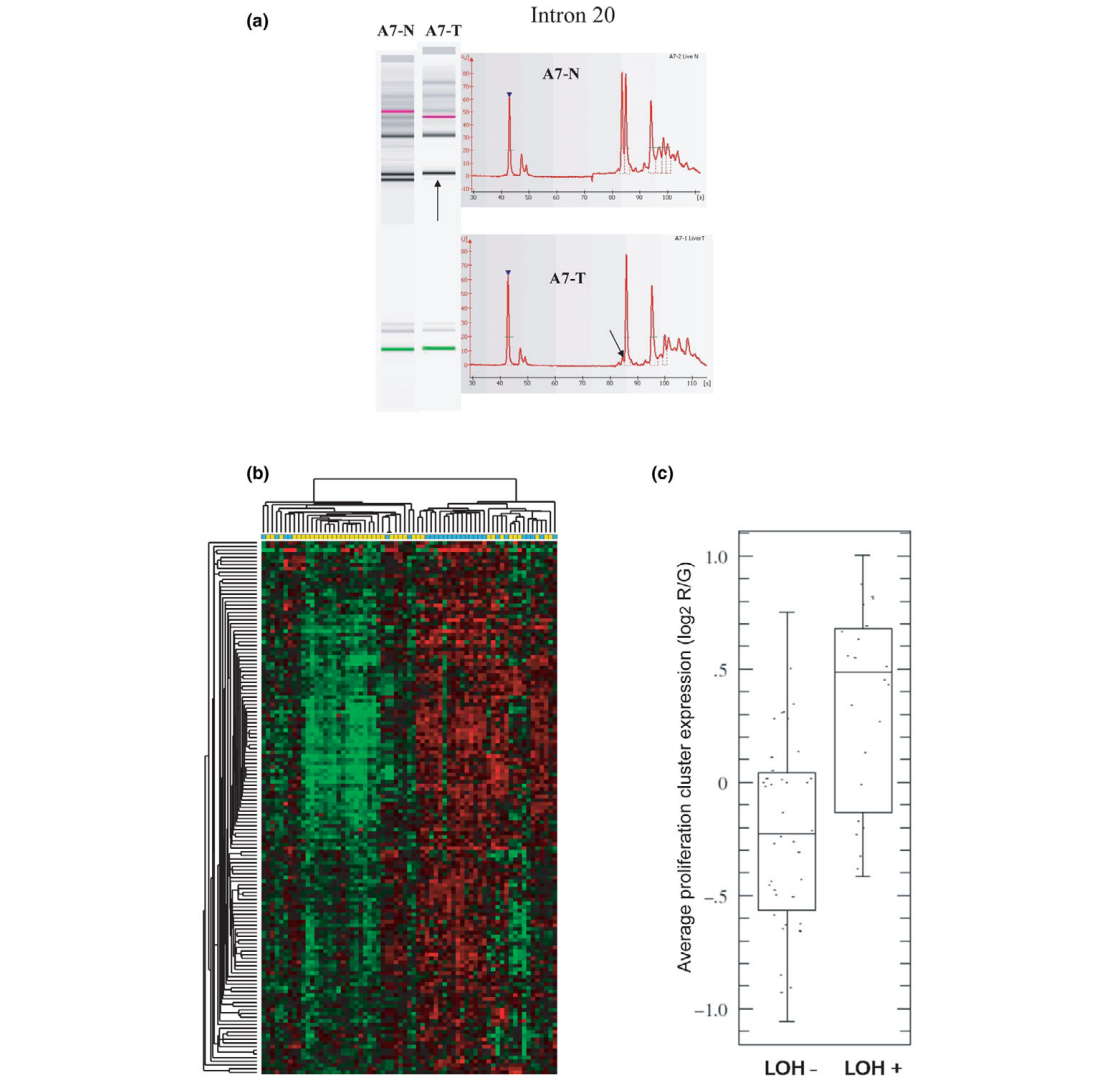
**Retinoblastoma (RB) 1 loss of heterozygosity (LOH) is associated with high proliferation**. (a) An example of LOH detected in a breast tumour sample at RB1 intron 20. The arrows point to the missing allele with A7-N being DNA from normal tissue and A7-T is tumour DNA from the same patient. (b) Two-way unsupervised hierarchical clustering of breast tumour samples with informative RB1 LOH status (LOH + = blue, LOH - = yellow) using the proliferation gene cluster. (c) Box plot comparison of the average proliferation cluster expression to RB1 LOH status.

Due to the low expression of RB1 message in basal-like tumours we decided to examine these breast carcinomas for LOH at the RB1 locus. We investigated 88 paired primary human breast carcinomas and normal tissue genomic DNA samples to assess the frequency of LOH in RB1. We used two polymorphic markers located at the RB1 locus (13q14); a variable number tandem repeat (VNTR) in intron 20 and D13S153, that is a microsatellite marker located within intron 2. There were 67 cases that were informative for at least one of these two markers (see Figure 3a as an example LOH). In total, 26 tumours showed RB1 LOH for at least one marker (26 of 67, 38.8%). This is consistent with the frequency of RB1 LOH seen in previous studies of breast cancer (26 to 47%) [5,6,25].

Next, using a previously defined proliferation gene signature [9], we clustered the gene expression data for this signature using just the 67 LOH informative patients (Figure 3b). This analysis was able to sort the samples into two groups with the right most group containing most of the RB1 LOH positive tumours (21 of 27, 77.8%). The left most group contained 29 of 40 (72.5%) of the RB1 LOH-normal tumours. It is important to note, however, that many of the LOH positive tumours that did cluster in the left group were located on the outer nodes and still showed higher expression of the proliferation markers than the tumours in the centre. Overall, an ANOVA showed that RB1 LOH was highly correlated with the high expression of the proliferation gene cluster (p = 0.0001) (Figure 3c).

We next classified the tumours that were assayed for RB1 LOH analysis according to intrinsic subtype using the five-class single sample predictor [9]. The frequency of RB1 LOH varied by molecular subtype (p = 0.0002) (Table 1). The lowest LOH frequencies were observed in the luminal A (3 of 20, 15%) and normal-like (0 of 7, 0%) subtypes, while the HER2-enriched subtype had a frequency near the breast cancer average (3 of 9, 33.3%). The highest frequency of RB1 LOH was observed in tumours of the basal-like (13 of 18, 72.2%) and luminal B subtypes (8 of 13, 61.5%), both of which are known to be highly proliferative tumour subtypes [9-11].

**Table 1:** Subtype specificity of RB1 LOH and RB1 and p16^INK4a ^immunohistochemistry

	**Basal-like**	**HER2-enriched**	**Luminal A**	**Luminal B**	**Normal-like**	**Total**	
**RB1 LOH**							
**LOH positive**	13 (48.2)	3 (11.1)	3 (11.1)	8 (29.6)	0 (0)	27	
**LOH negative**	5 (12.5)	6 (15.0)	17 (42.5)	5 (12.5)	7 (17.5)	40	
**Total**	18	9	20	13	7	67	p = 2.26E-04
							
**RB IHC**							
**0**	6 (42.9)	2 (14.3)	3 (21.4)	3 (21.4)	0 (0)	14	
**1+**	13 (29.6)	5 (11.4)	14 (31.8)	4 (9.1)	8 (18.2)	44	
**2+**	5 (12.8)	6 (15.4)	14 (35.9)	6 (15.4)	8 (20.5)	39	
**3+**	7 (35.0)	2 (10.0)	4 (20.0)	7 (35.0)	0 (0)	20	
**Total**	31	15	35	20	16	117	p = 0.0644
							
**p16 IHC**							
**0**	2 (8.0)	4 (16.0)	8 (32.0)	6 (24.0)	5 (20.0)	25	
**1+**	5 (15.2)	3 (9.1)	15 (45.5)	5 (15.2)	5 (15.2)	33	
**2+**	4 (13.8)	4 (13.8)	9 (31.0)	6 (20.7)	6 (20.7)	29	
**3+**	22 (68.8)	4 (12.5)	3 (9.4)	3 (9.4)	0 (0)	32	
**Total**	33	15	35	20	16	119	p = 1.41E-05

0 = negative, 1+ = weak positive, 2+ = moderate positive, 3+ = strong positive. P-values determined by Fisher's exact test.

HER2 = human epidermal growth factor receptor 2; IHC = immunohistochemistry; LOH = loss of heterozygosity; RB1 = retinoblastoma.

### RB1 and p16^INK4a ^immunostaining in breast carcinomas

**Figure 4:**
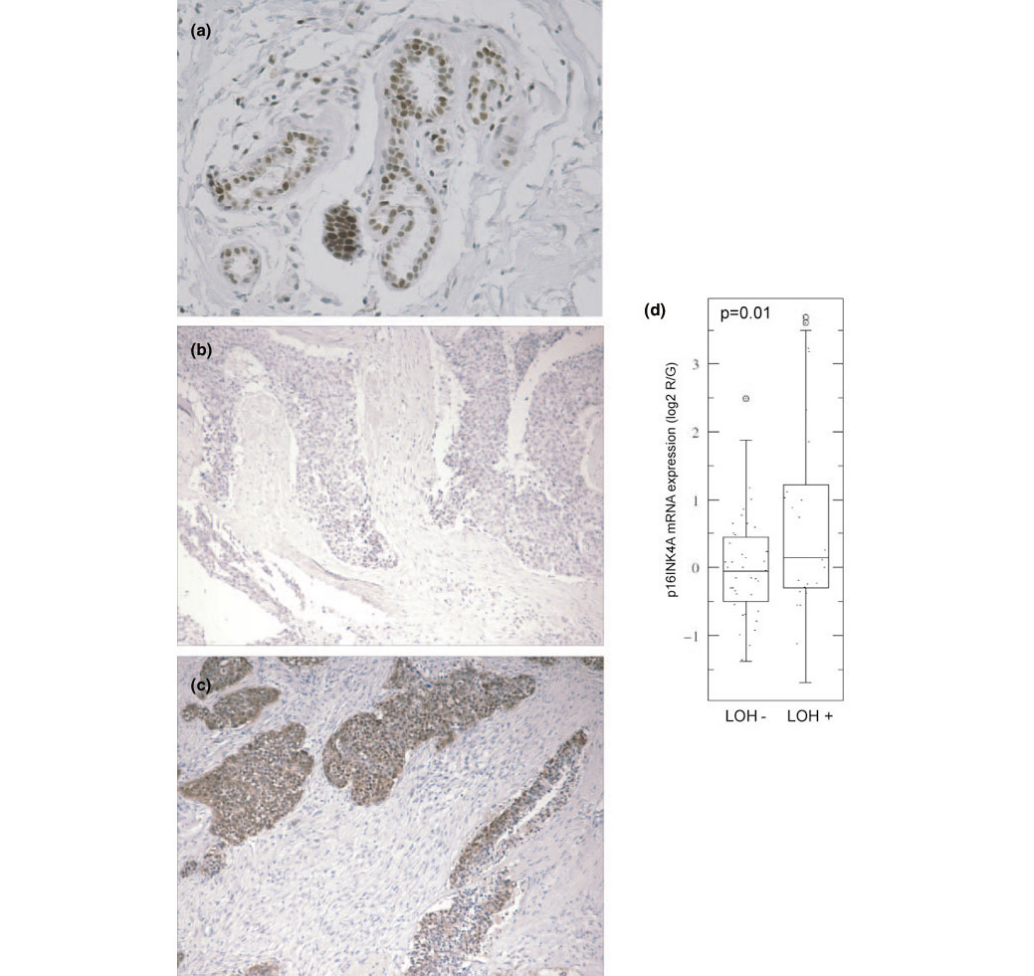
**High p16^INK4a ^mRNA and protein levels are associated with retinoblastoma (RB) 1 loss of heterozygosity (LOH)**. (a) RB1 staining of normal breast tissue, (b) RB1 LOH+ basal-like tumour lacking RB1 staining, and (c) the same RB1 LOH+ basal-like tumour showing staining for p16^INK4a ^both nuclear and cytoplasmic.**(**d) Box plot comparison showing high p16^INK4a ^mRNA expression in RB1 LOH + breast tumours.

RB1 is expressed ubiquitously in mammary epithelial cells and typically shows a nuclear staining pattern in normal human breast tissue (Figure 4a). RB1 immunostaining was statistically correlated with RB1 message levels (p = 0.0081) but, as has been described before, RB1 protein expression did not correlate with RB1 LOH (p = 0.5) [6]; however, a trend for low expression of RB1 message to occur with RB1 LOH was observed (p = 0.11). RB1 protein expression also tended to be low in basal-like tumours (Figure 4b and Table 1), but this relationship was only near statistical significance (p = 0.064).

High p16^INK4a ^staining (3+), which is a hallmark of lost RB1 function [26,27], was seen in 32 of 119 tumours assayed and often included both nuclear and cytoplasmic staining (Figure 4c). p16^INK4a ^immunostaining was statistically correlated with p16^INK4a ^message levels (p = 0.013), especially when high staining was observed (data not shown). p16^INK4a ^immunostaining was also associated with intrinsic tumour subtype (p = 1.41E-05) with 22 of 33 (66.7%) of basal-like tumours showing 3+ staining (Figure 4c, Table 1). The correlation between RB1 LOH status and p16^INK4a ^expression levels was statistically significant (p = 0.01) (Figure 4d, Table 2). In addition, p16^INK4a ^was similarly highly expressed in transgenic murine mammary tumours with loss of RB function driven by SV40 large T-antigen or T121 (Additional File 1) [14]. When considered together, these data suggest that RB1 LOH in basal-like tumours cause a loss of RB1 protein function, which the cells attempt to compensate for by increasing p16^INK4a ^gene and protein expression levels.

**Table 2:** Comparison of p16^INK4a ^immunohistochemistry and RB1 LOH status

	**p16 IHC 0**	**1+**	**2+**	**3+**	**Total**	
**RB1 LOH**						
**LOH-**	9 (23.7)	11 (29.0)	11 (29.0)	7 (18.4)	38	
**LOH+**	1 (3.9)	8 (30.8)	5 (19.2)	12 (46.2)	26	
	10	19	16	19	64	p = 0.0369

0 = negative, 1+ = weak positive, 2+ = moderate positive, 3+ = strong positive. P-values determined by Chi-square test.

IHC = immunohistochemistry; LOH = loss of heterozygosity; RB1 = retinoblastoma.

### RB1 LOH gene expression signature

To determine if there was a gene expression signature related to RB1 LOH, a two-class SAM [20] was performed of RB1 LOH positive tumours vs. LOH-normal tumours. In total there were 452 genes that varied with RB1 LOH status with a false discovery rate of 0.94%, as compared with 11 genes identified at a false discovery rate of 16.7% when a similar analysis was performed using RB immunostaining data (0 vs. 1, 2 and 3). Of these genes, 423 were highly expressed in tumours with RB1 LOH and an analysis of the gene ontologies associated with this gene set showed that cell cycle, cell division, DNA metabolism, spindle organisation and biogenesis, and response to DNA damage were the top biological processes discovered when using Bonferroni-corrected scores. Interestingly, E2F1, E2F3 and E2F5 were present on this supervised gene list and highly expressed in tumours with RB1 LOH. Also present in this list was RB1CC1, a regulator of RB1 expression that has been shown to contain truncating mutations in breast cancers [28]. We used whole genome RVista to calculate which transcription factor binding sites might be present within the 1000 bp upstream regions of these genes [21] and determined that the top three transcription factor binding sites with p < 0.005 were E2F4:DP1, E2F1:DP1:RB and E2F4:DP2, showing that a majority of these genes are likely to be E2F-regulated; other statistically significant transcription factor binding sites were HIF1:ARHN. Only 29 genes were significantly negatively/downregulated from the RB1 LOH SAM analysis, and there were no significant gene ontology categories enriched in this list.

### RB1 LOH gene expression signature correlates with signatures of proliferation and RB-loss

**Figure 5:**
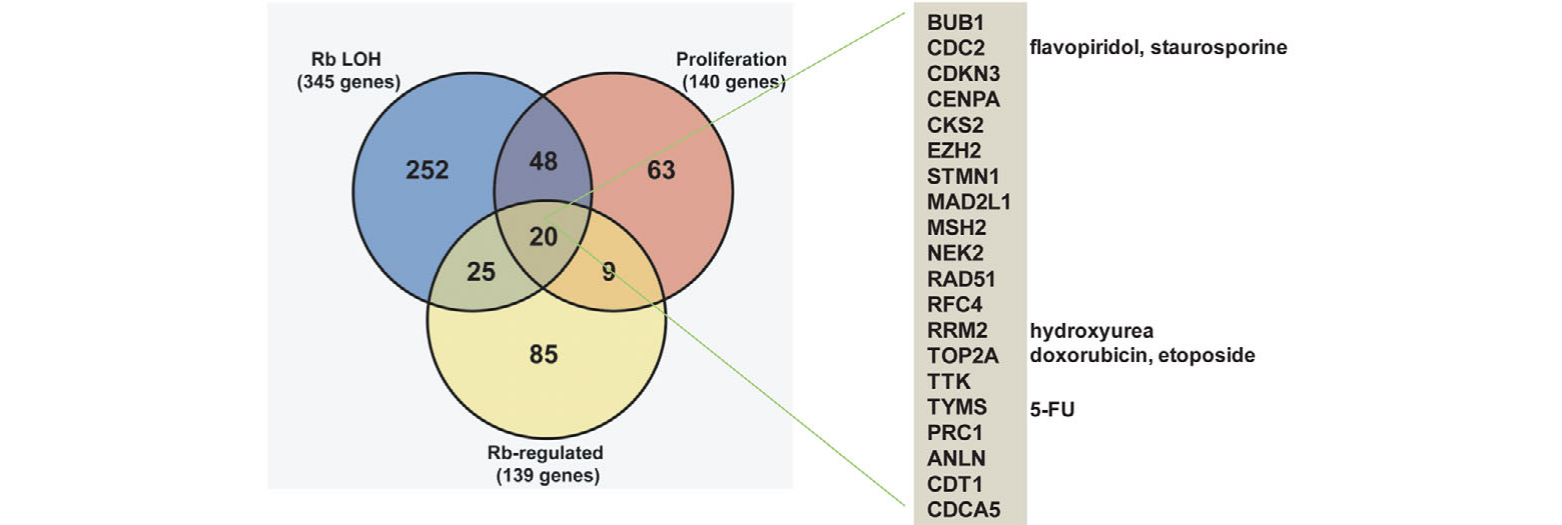
Venn diagram showing the overlap between the retinoblastoma (RB) 1 loss of heterozygosity (LOH), proliferation, and RB1-loss gene lists.

Recently, an RB-loss gene expression signature was derived using mouse fibroblasts with either acute or chronic knockout of RB1 using conventional knockout as well as Cre-Lox technology [29]. As might be expected, this RB-loss signature was highly expressed in basal-like tumours (Additional File 2). This RB-loss signature significantly overlapped with the proliferation signature (29 of 139 genes of the RB-loss signature are contained in the 140 gene human proliferation signature used here, hypergeometric mean p < 0.001), thus serving as further evidence that the proliferation signature contains many RB1-E2F regulated genes. There was statistically significant overlap among all three RB-pathway signatures studied here (ie, RB-LOH, RB-loss and proliferation) as determined by hypergeometric mean analysis p < 0.001, and thus all three signatures are probably tracking a common biology that is RB1-E2F dependent. There were 20 genes that overlapped between all three gene lists (Figure 5), which included cell cycle-related genes including the spindle assembly checkpoint proteins BUB1 and MAD2, and many commonly used chemotherapeutic drug targets including TOP2A (doxorubicin, etoposide), thymidylate synthetase (5-FU), ribonucleotide reductase M2 (hydroxyurea) and CDC2 (flavopiridol, staurosporine).

**Figure 6:**
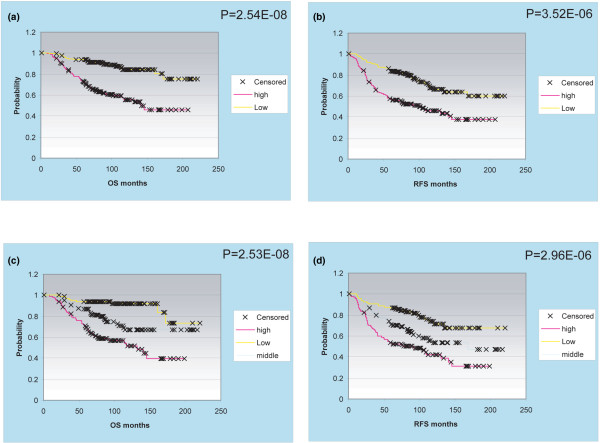
**Retinoblastoma (RB) 1 loss of heterozygosity (LOH) gene list is highly predictive of breast cancer patient outcome**. Kaplan-Meier survival curves showing overall survival (OS) or relapse-free survival (RFS) by dividing the patients in thirds (a, c) or in halves (b, d) using the rank order average expression values of the RB-LOH signature using the NKI295 breast cancer data set.

All four of these RB-pathway associated lists (RB-LOH, RB-loss, proliferation signature and the 20 common genes) were highly predictive of breast cancer patient outcomes when using a two-class or three-class, average value rank order expression cutoff and when tested on the NKI295 patient data set (Figure 6, shown using RB-LOH list and data not shown for the other three lists that give very similar results) [30], and on a previously described 251 patient data set (data not shown) [31]. It should be noted, however, that several groups have independently identified different gene lists that contain a large number of so-called proliferation/RB-pathway genes [13,32-35] and it was expected that the RB-LOH signature would be a strong prognostic profile.

The RB-pathway associated signatures were predictive of poor prognosis in breast cancers. But are they predictive of response to therapy? To answer this question, we determined if the RB-LOH signature correlated with pathological complete response in a well-annotated data set of neoadjuvant chemotherapy-treated patients [36]. The study by Hess and colleagues included 133 patients with stage I to III breast cancer that were treated with preoperative weekly paclitaxel followed by fluorouracil-doxorubicin-cyclophosphamide chemotherapy. High expression of each of the four RB-pathway signatures was associated with pathological complete response in the breast and regional lymph nodes (Additional File 3). Thus, high expression of the RB-LOH and proliferation signatures is associated with a good response to neoadjuvant chemotherapy in breast cancers.

## Discussion

Our understanding of breast cancer biology has been improved by the identification of genomically defined tumour subtypes. These subtypes are defined by distinct gene expression patterns, molecular changes and potentially distinct developmental cell types of origin adding up to observed differences in outcome and responses to therapy. In this report, we show that the frequency of RB1 LOH varied significantly according to 'intrinsic' subtype. RB1 LOH occurs at a frequency of 72.2% in basal-like breast tumours and 61.5% in luminal B tumours, both of which are observed in retinoblastomas in the frequency range of 60 to 75% [37-40]. RB1 protein staining as assessed by immunohistochemistry, however, did not correlate with RB1 LOH in our study (as has been reported before); however, a SAM analysis supervised by RB1 LOH robustly identified many E2F genes, and E2F-regulated target genes, while the RB1 protein-guided SAM analysis did not. This suggests that RB1 LOH is a better biomarker of RB-pathway function than immunohistochemistry staining for total RB1 protein.

Additional support for the functional loss of RB1 in basal-like tumours comes from the correlation with high p16^INK4a ^message and protein expression. The inverse relationship between p16^INK4a ^and RB1 expression in breast cancers has been previously reported [41,42]; however, this relationship and its association with basal-like tumours is new in this report. Another intriguing link between p16^INK4a ^and basal-like cells comes from studies on human mammary epithelial cells, which have been shown to resemble the basal-like subtype by gene expression analysis [43,44]. It has been shown that in order for human mammary epithelial cells to proliferate *in vitro *in culture for an extended period they must overcome an RB-mediated stress associated senescence barrier (stasis), which usually involves spontaneously losing p16^INK4a ^expression by promoter methylation [45]. The gene expression changes associated with this *in vitro *transition are similar to those we previously reported to occur with RB1 LOH [46].

In basal-like tumours *in vivo*, however, the exact opposite seems to occur in that the RB-pathway barrier appears to be RB1 functional loss with a concomitant feedback loop that induces p16^INK4a ^gene and protein expression. The link between high p16^INK4a ^expression being caused by RB1 loss is known; RB1 recruits Polycomb repression complexes to the p16^INK4a ^locus, which silence p16^INK4a ^transcription [47]. It is also well-known that cell cycle inhibition by p16^INK4a ^is RB-dependent [48] and, therefore, these RB1-deficient breast tumours would be expected to be refractory to the high levels of p16^INK4a^. This explains their high proliferation rates in the presence of high levels of p16^INK4a^. High p16^INK4a ^expression has reproducibly been shown to be associated with poor prognosis [41,49-51] and in a recent study by Grupka and colleagues, p16^INK4a ^staining of sentinel lymph nodes was predictive in determining the presence of non-sentinel node metastases [52]. Gauthier and colleagues have recently shown that in ductal carcinoma *in situ *(DCIS) lesions, high p16^INK4a ^together with low Ki-67 (proliferation) appears to not promote tumour progression, while high p16^INK4a ^and high Ki-67 lead to subsequent tumours [24]. Lastly, it has been demonstrated that the deletion of RB1 in the murine mammary gland is capable of initiating tumourigenesis and that many of these resulting tumours have basal-like features (E. Zacksenhaus, personal communication). In total, these data strongly argue that the RB-pathway lesion that occurs in most basal-like tumours is RB1 loss, possibly with a compensatory activation of p16^INK4a^.

Basal-like tumours also highly express a recently published RB-loss gene expression signature [29,32], which we have shown to have significant similarity to a previously defined proliferation signature and our newly described RB1 LOH signature. Lastly, the p16^INK4a ^expression seen in DCIS by Gauthier and colleagues, and the elevated Ki-67 index seen in basal-like DCIS lesions, suggests that RB1 loss may be an early event for this tumour type [53].

Similar to basal-like tumours, luminal B tumours also showed a high frequency (61.5%) of RB1 LOH in our study, but this was not associated with induction of p16^INK4a^. The differential effect of RB1 loss on p16^INK4a ^expression in luminal B versus basal-like tumour cells implicates other transcription factors in addition to pRb-E2F in the regulation of this CDK inhibitor in luminal tumours. In the recent study by Bosco and colleagues, luminal tumour-derived cell lines were shown to be more proliferative and resistant to hormone therapy after knockdown of RB1 [32], both of which are signatures of luminal B tumours [9-11]. The RB-loss signature was shown to be predictive of outcome in a data set containing only oestrogen receptor positive (ER+) breast tumours treated with tamoxifen monotherapy. Therefore, the loss of RB1 function may also play a substantial role in the increased proliferation, possible resistance to hormonal therapies and poor prognosis that is seen in luminal B tumours. In addition, the knockdown of RB1 in established breast cancer cell lines has recently been shown to increase sensitivity to a variety of DNA-damaging therapeutic agents [32,54]. While these experiments were performed with ER+ tumour cell lines, it does open the possibility that the RB1-defect in basal-like tumours plays a role in their increased chemosensitivity compared with most luminal tumours [55,56]. This is supported by our findings of increased neoadjuvant response in patients expressing high levels of the four different RB1-proliferation associated signatures.

The presence of LOH is typically thought to indicate that a mutated allele is present on the other chromosome and that the LOH makes the cell homozygous or hemizygous for the mutated allele. There is little published evidence to suggest that dramatic structural changes aside from LOH are occurring at the RB1 locus in breast tumours. There are a few reports of alterations in RB1 in breast cancer with two reports showing structural changes as assessed by Southern blotting in 7% and 19% of primary tumours [57,58], and no published reports to our knowledge of point mutations. Interestingly, a study by Kallioniemi and colleagues looking at RB1 loss in clinical breast cancer samples by fluorescent in situ hybridisation showed that most of the cells within these tumours contained two copies of the RB1 gene even when they showed LOH by restriction fragment length polymorphism at the RB1 locus [59]. When these studies are considered with the data presented here, they suggest a complex scenario where one allele is lost by LOH and the remaining allele/residual protein is compromised by an as yet to be identified mechanism(s) that potentially varies between tumour subtype, and potentially varies even within basal-like tumours. For example, some basal-like tumours with LOH show complete loss of RB protein, while others show expression and both types show high proliferation. As opposed to breast cancer, there is a great deal known about the mechanisms of RB1 loss in retinoblastoma [60-62]. As in retinoblastoma, it is clear that a combination of techniques will need to be applied in order to identify the precise mechanisms of RB1 inactivation in breast cancer.

## Conclusion

We have shown that RB1 LOH is a frequent occurrence in basal-like and luminal B breast tumours and is associated with deregulation of E2F-regulated genes. Deregulation of the RB-pathway in cell lines has shown that it may be an important determinant of response to therapy [32]. Moreover, we have demonstrated that RB1 loss signatures can be used to predict neoadjuvant chemotherapy response. Therefore, RB1 function may be an important biomarker for informing treatment decisions.

## Abbreviations

ANOVA: analysis of variance; CDK: cyclin dependent kinase; DCIS: ductal carcinoma in situ; ER: oestrogen receptor; GEO: Gene Expression Omnibus; HER2: human epidermal growth factor receptor 2; LOH: loss of heterozygosity; PBS: phosphate-buffered saline; PCR: polymerase chain reaction; RB1: retinoblastoma; SAM: Significance Analysis of Microarrays; TP53: tumour protein p53; VNTR: variable number tandem repeat.

## Competing interests

CMP has stock ownership in University Genomics. Other authors declare no competing interests.

## Authors' contributions

JIH participated in the data analysis, scoring of immunohistochemistry staining, development of the figures and the writing of the manuscript. XH prepared DNA from tumours and normal patient lymphocytes, and immunohistochemistry staining and scoring. CF performed statistical analysis. CMP was the principal investigator, conceived and designed the study, and drafted the paper. All authors read and approved the final manuscript.

## Supplementary Material

Additional File 1 A PDF file containing a figure comparing p16^INK4a ^and proliferation gene expression across 13 transgenic murine mammary tumour models. Click here for file

Additional File 2 A PDF file containing a figure showing a box plot comparison of RB-loss signature relative to the five intrinsic subtypes as defined by the 5-class centroid predictor. Click here for file

Additional File 3 A PDF file containing a figure showing a box plot comparison of average expression of a) RB-LOH, b) RB loss, c) proliferation signature and d) 20-gene overlap signature relative to response to neoadjuvant treatment. Click here for file
