# Supplementary figures and images for: The functional loss of the retinoblastoma tumour suppressor is a common event in basal-like and luminal B breast carcinomas
Source: Breast Cancer Res. 2008 Sep 9;10(5):R75. doi: 10.1186/bcr2142 (PMC2614508; doi:10.1186/bcr2142)

# Herschkowitz\_Additional Data File 1

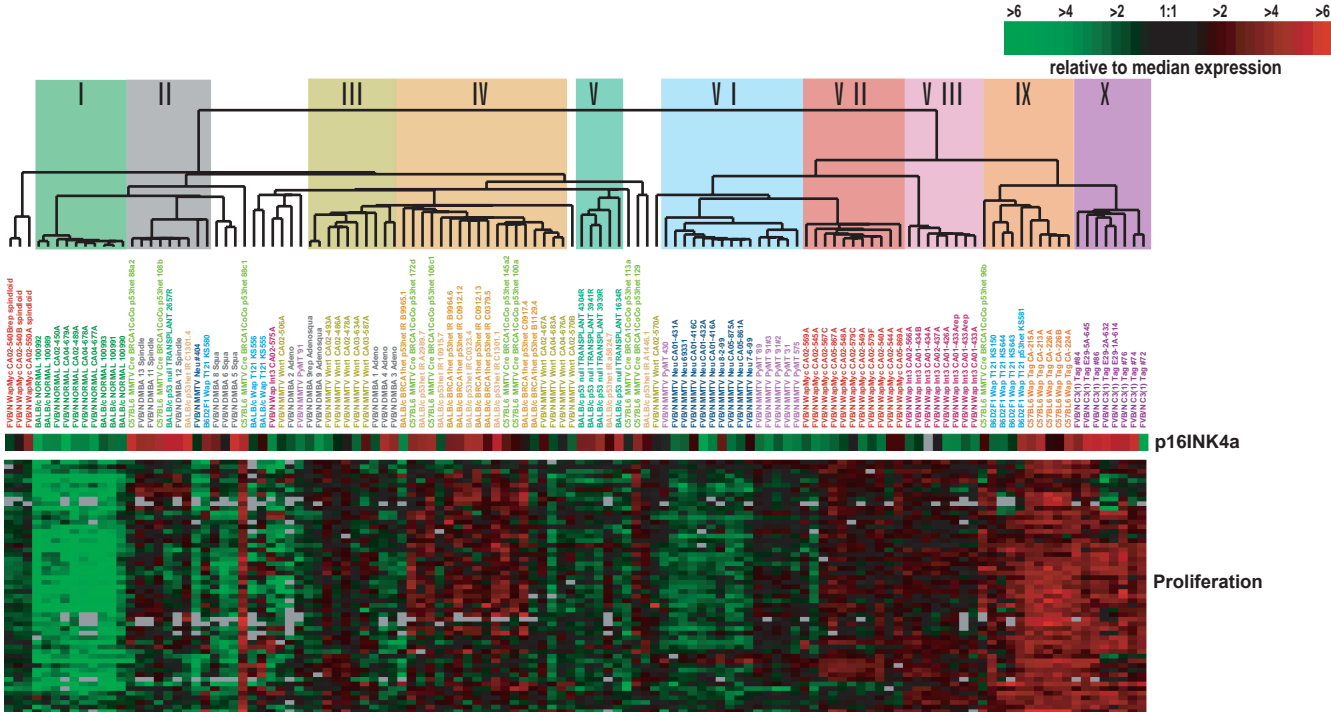

Supplement: Additional File 1 — A PDF file containing a figure comparing p16INK4a and proliferation gene expression across 13 transgenic murine mammary tumour models. [file bcr2142-S1.pdf]

## Herschkowitz\_Additional Data File 2

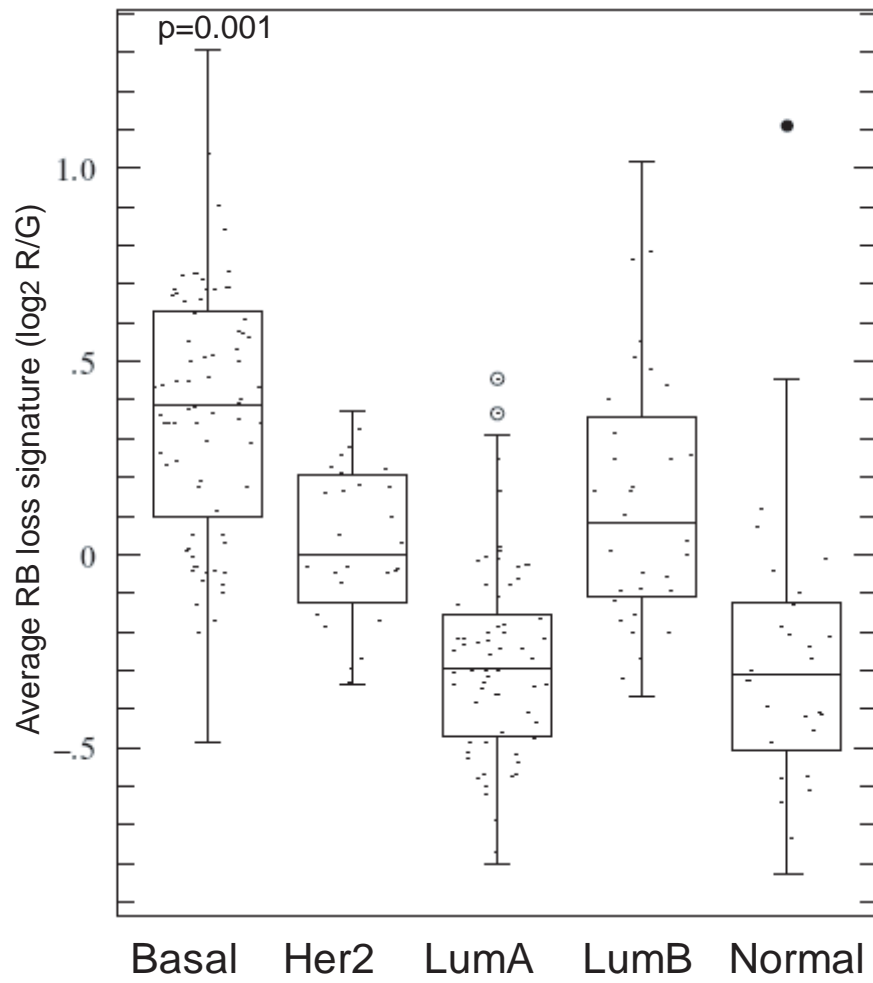

Supplement: Additional File 2 — A PDF file containing a figure showing a box plot comparison of RB-loss signature relative to the five intrinsic subtypes as defined by the 5-class centroid predictor. [file bcr2142-S2.pdf]

# Herschkowitz\_Additional Data File 3

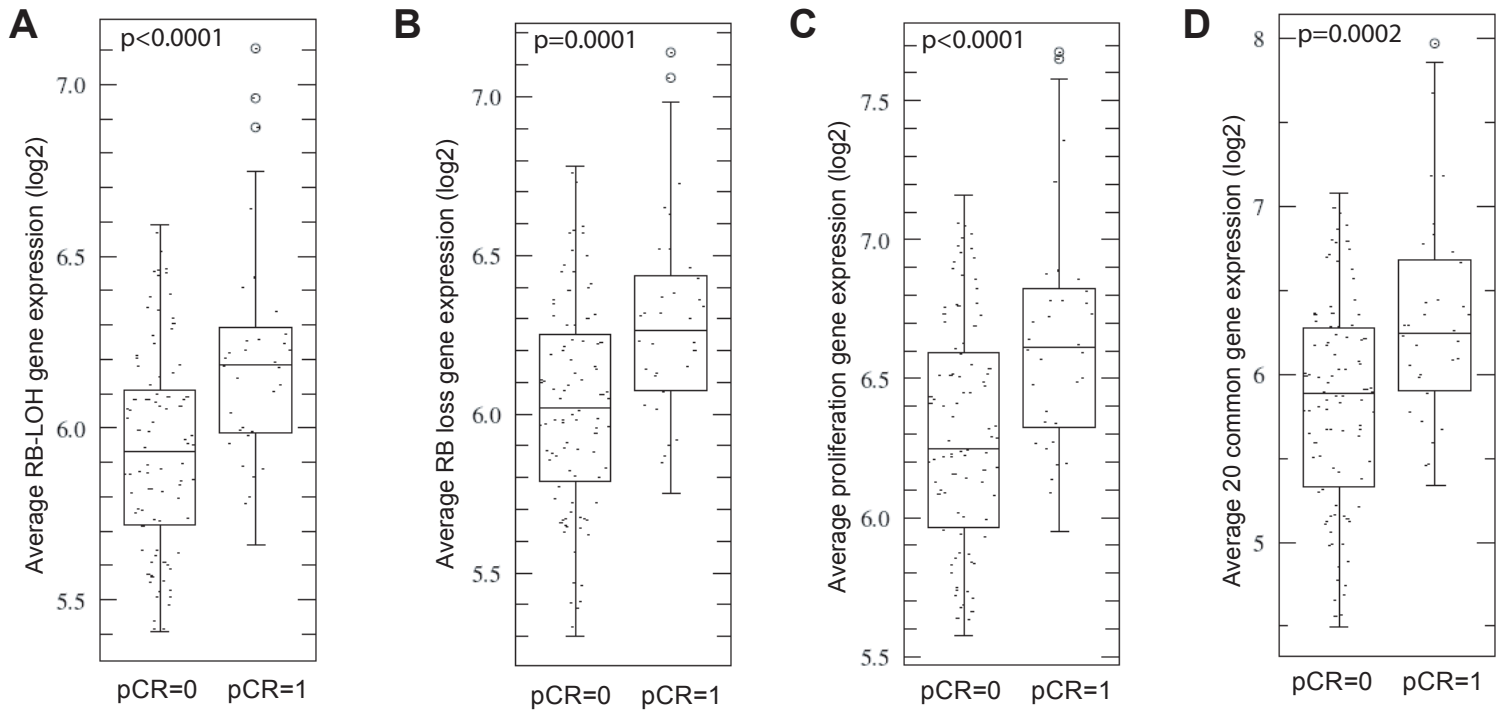

Supplement: Additional File 3 — A PDF file containing a figure showing a box plot comparison of average expression of a) RB-LOH, b) RB loss, c) proliferation signature and d) 20-gene overlap signature relative to response to neoadjuvant treatment. [file bcr2142-S3.pdf]
